# The Prevention of Disease

**Published:** 1893-08

**Authors:** DeLancey Rochester

**Affiliations:** Adjunct Professor of Medicine in the University of Buffalo; Assistant Visiting Physician to the Buffalo General Hospital


Buffalo Medical and Surgical Journal

Vol. XXXIII. AUGUST, 1893. No. 1.

### Original Address.

### THE PREVENTION OF DISEASE.

By DeLancey Rochester, M. D.,
Adjunct Professor of Medicine in the University of Buffalo; Assistant Visiting Physician to the Buffalo General Hospital.

PRESIDENT'S ADDRESS BEFORE THE BUFFALO ACADEMY OF MEDICINE.
ANNUAL MEETING, JUNE 27, 1893.

A little over a year ago, at a meeting of physicians called for the purpose of forming the Clinical Society, some remarks were made deprecating the formation of another medical society in Buffalo, and suggesting that, instead of such action, we concentrate our energies in the Buffalo Medical and Surgical Association, dividing it into sections after the manner of the New York Academy of Medicine. The idea was not new, but that the time was ripe for its germination was proved by the general favor with which the suggestion was received. However, after full and free discussion, it was deemed best not to concentrate in the Buffalo Medical and Surgical Association, but to invite several other societies to coöperate in the effort to concentrate the scientific medical work that was being done in the city, in the manner suggested. Accordingly, a committee was appointed by the newly-formed Clinical Society to confer with committees which might be appointed by other societies, as to the advisability and practicability of the action suggested. Committees were appointed by the Buffalo Medical and Surgical Association, by the Pathological Society, and by the Obstetrical Society, to confer with the committee of the Clinical Society. These committees had several meetings, and finally, as a result of these conferences, a joint meeting of the several societies was held, at which it was decided to unite in the formation of the Buffalo Academy of Medicine, a constitution was adopted, and the officers under that constitution were duly elected. So far all seemed well, but the members of the various societies did not, with alacrity, complete their membership by the payment of initiation fees and annual dues, seeming to doubt the virility of the new Academy, and not being willing to aid in its nurture during its infancy ; so that, at the first stated meeting of the Buffalo Academy of Medicine, there were but thirty names enrolled upon the membership list ; to-day there are one hundred paid-up members! Our first meetings were held in rooms, hired for the evening, here and there in different parts of the city; to-day we have spacious apartments to which we are proud to invite visitors from out of town ; we have, through the kindness of Dr. Bartlett, the beginnings of a library, and we are starting a pathological museum. Truly, I think we may congratulate ourselves upon the growth of the Academy. Moreover, the stability of the Academy is assured, from the fact that this growth is in no respect the result of the energy of any one man, but is the outcome of the combined energy of all.

Nevertheless, it is but just to say that this energy was fired into activity by the enthusiasm and self-sacrificing public spirit of Dr. Herman E. Hayd, who has thrown himself with so much vigor into the work of increasing the membership and raising money by subscription for furnishing our apartments, that the rest of us have been forced for very shame to follow his noble example. No factional fights have so far disturbed the Academy, and it is to be sincerely hoped that nothing will ever arise to disturb the harmony of this new scientific body.

As it fell to my lot to be the lucky one to suggest the formation of the Academy, you decided to make me its first president, and thus, to a certain extent, to throw the responsibility for its success or failure upon my shoulders. I take this opportunity to thank you all for the aid you have given me to bear this responsibility. Truly, I think we may congratulate each other upon the bright auspices under which it begins its second year. The constitution requires an annual address from the retiring president ; it is my desire to inaugurate the custom of making the subject of that address, one of general medical interest that may *also* be of value to the community in which we live, and I have accordingly chosen as the subject of my address this evening Practical Measures Tending to the Prevention of Disease.

It has been said—and is constantly being re-asserted—that the medicine of the future is to be preventive medicine. When is the "future" to begin ? How much has any of us done to aid the advent of that most desirable period ? Moreover, what are we to do to accomplish so laudable a purpose ? As far as I can learn from personal observation and from careful study of the records of the past, I am sorry to say that most physicians have shamefully neglected their evident duty to humanity by contenting themselves with merely aiding in the recovery of a patient from special disease or condition, and then dismissing the case from further consideration. Now, it seems to me that it is the duty of the physician—nay, he should esteem it a most blessed privilege—to be more than a mere healer of the sick, to be an *educator* of his fellow-men to such extent that they may learn to so physiologically live as to avoid disease and postpone death. In order thus to teach, one must have thorough knowledge one's self. The first requisite, then, for the much-desired preventive medicine is that all physicians should be thoroughly educated men or women ; the standard of medical education must be raised to the highest degree.

While it is undoubtedly true that there have been some excellent physicians who have not received an academic degree before beginning the study of medicine, I think it is also true that they have attained their knowledge at great sacrifice *after* they have graduated in medicine, and might have attained their eminence at a much earlier period if their previous education had been broader.

It is generally conceded that a man should not try to become a specialist in any branch of medicine until he has had a solid foundation of several years of practice in general medicine.

Is it not equally true that no man should devote himself to any one branch of science, such as medicine, until after he has been *thoroughly* founded in general knowledge—arts, sciences, languages, and philosophy ?

The first step, then, towards the attainment of preventive medicine, is the requirement that all who desire to *begin* the study of medicine should possess an academic degree or present proof of equivalent preliminary education. The next step is the requirement of a ninety per cent. standard for graduation.

Some members of some faculties seem not to realize that, when they allow incompetent or unworthy men to graduate from their medical schools, by signing the diploma of such a one, they are making themselves, in fact, if not in law, accessory to any crime or evil that may be brought about or occur through the wickedness or ignorance of such a graduate. Let us, then, do all we can to force the medical schools to raise their standard for admission and for graduation. Then, indeed, we will hear less of exclusive systems of practice, secret and patented medicines and other forms of charlatanry; and a medical profession, composed of highly educated members, will be able to truly educate the public as to a mode of life that will tend to prevent the development of disease.

In order to bring about the end desired, every one of us, whose privilege it is to occupy the position of family physician, should begin the work of education with the mother of the unborn babe; he should carefully watch over her through her pregnancy, advise her as to every detail of her daily life, especially as to exercise, diet, and the functions of excretion, encourage her to ask him about anything on which she wishes enlightenment, and make her feel that in him she has a friend who will lend a sympathetic ear to anything she wishes to say, no matter how trivial it may appear.

Thus with a mother in good health through her pregnancy, the chances that the child born to her will be healthy, and that she will be able to nurse her child, are proportionately great. It is likewise our duty to insist on such a mother's nursing her child; and if we have done our duty, we will have so impressed the mother with the beauties and blessings of maternity, that she will rejoice to be able to nurse her baby. When the mother is up from her confinement, we should not cease our interest in the welfare of her and her child, but should give instruction as to when and how to wean, what to feed the child and *how* to feed it. Thus the child will be started on the road to health.

Now, it is our duty to give instruction, whenever we have the opportunity in a public way, and to all of our families, in general hygienic laws, such as proper ventilation, the importance of regularity about meals, defecations and hours of sleep—as regards meals, we should also give instruction as to what foods are incompatible and should not be eaten at the same meal, and as to the importance of the function of proper mastication.

In our system of school education should be included an elementary course in human physiology, in which every child should be instructed before he or she has reached the age of fourteen or fifteen years. The instructor in physiology should always be a thoroughly educated physician.

So much, then, as regards general instruction for the production and maintenance of health.

Let me call your attention for a few minutes to certain measures for the prevention of special diseases. Although I believe that the acute infectious diseases, diphtheria, scarlatina. measles, rötheln roseola, varicella, variola, and whooping-cough, could be absolutely stamped out by the passage of stringent laws and the thorough enforcement of the same by the municipal authorities against both physicians and householders, I will not take up that subject this evening. There is a greater danger than that from all these diseases combined, which has menaced for years, and continues to menace, the human race; there are certain diseases which carry off millions of people and greatly debilitate others, which are generally incurable when contracted, but could be absolutely avoided if the people only knew how. It is our duty to instruct the people how to avoid these diseases, among which tuberculosis is *facile princeps* in the death rate, and the venereal diseases, syphilis and gonorrhea, are not far behind in the debilitating effect upon the individual and his progeny. Let me call your attention to some measures which we might adopt to prevent the spread of these fearful scourges. For the prevention of a disease by any rational measures, the cause or causes of such disease must be thoroughly understood. After Koch had proved that the bacillus tuberculosis was the cause—the sole active cause—of pulmonary consumption, our hopes ran high that now that we had a definite foe to attack, it would not be long before victory should perch upon our standards and that fell destroyer of the human race would be driven from the face of the earth forever.

But we have learned that once the bacilli have gained entrance into an organism, it is seldom that they can be evicted.

"The destruction of organisms outside of the body by disinfectants," says Flint, Sr., "and the removal of all the accessory conditions necessary to their existence, together with their effectual exclusion from the body, may, and indeed it is more than probable will, reduce the fatality from infectious diseases, in the order of Providence, much sooner than the discovery of effective therapeutic agents."

Among the "accessory conditions necessary to their existence" we find that there are certain types of individuals more susceptible than others to the attacks of the tubercle bacilli. The old writers laid a great deal of stress upon the recognition of different natural temperaments in healthy individuals, that modified the course of disease in such individuals, and upon certain pathological diatheses, that rendered those possessing them more liable to certain diseases.

It is not my purpose to take up this most interesting subject further than to call your attention to the fact that there is a certain physical condition, that renders its possessor more susceptible to the onslaught of the tubercle bacillus, that this condition is marked by certain physical characteristics, and that it is sometimes inherited and sometimes acquired.

It is not necessary here to enumerate those characteristics ; I wish merely to point out the fact that they are, almost without exception, due to malnutrition accompanying or accompanied by anemia of considerable degree.

If this is so,—and I think the experience of each of you will bear me out in the statement that it is,—one of the prime factors to be taken into account in the prevention of consumption, is the prevention of the preceding anemia. How is this to be done? In the first place, as far as in our power lies, we should discourage the marriage of any who has a hereditary taint.

As regards this, however, the physician is seldom consulted. Nevertheless, in his public utterances, he should do all he can to put this matter clearly before the people.

In other respects, the following of the suggestions already made in regard to general hygienic living will go far towards the prevention of the anemia. One of the great factors in the causation of anemia is a deficient supply of oxygen ; not that there is not sufficient oxygen about us, but a large number of individuals—especially those who have any hereditary tendency to consumption—do not know how to breathe, and so deprive themselves of an element that Nature supplies abundantly, which is so necessary to their welfare. Let us, then, teach people how to breathe, combining with the respiratory exercises the movements of the arms and legs, and abdominal muscles that will aid in the development. and expansibility of the chest. No apparatus is necessary for this Pay great attention to their excretory organs, the bowels, the liver, the skin, and the kidneys, and to their organs of digestion, the stomach, and the small intestine.

So much for hints as to how to prevent the anemia. If the anemia is present, treat it, but treat it intelligently, not by the mere pouring in of iron, but by careful examination of the patient in toto and by the combination with the iron of alkalies, oxygen by inhalation, phosphorus, arsenic, iodine, or cod liver oil, as may be indicated by the result of your examination.

So much for instruction how to avoid that physical condition which affords so excellent a culture medium for the development of tubercle bacilli.

Now, what can we do to prevent the entrance of the bacillus into the person of a healthy individual?

About two months ago, I was called to see a child dying of consumption. She had been under the care of another physician for several years. He had properly diagnosed her case and realized her hopeless condition. I confirmed his prognosis, and while giving the parents the sad information I took occasion to impress upon them the contagiousness of the disease and wherein the contagion lay, the importance of destruction of sputum, and avoidance of inhaling the child's breath, etc.

The child died that night, and the next day the mother told me that she had not previously observed much precaution as to the exhalation, sputum, and excretions of her child, because only the week before, she had asked the attending physician if there were any reason why her other child should not sleep in the same bed with the sick one, as some of her neighbors had warned her in that regard, and he had told her that there was no harm in it except that the well one might be disturbed by the restlessness of the sick one!

Is it not time that the people should be instructed by those who know, when those who do not know are allowed to practise medicine?

What instructions, then, should we give to those who are suffering from phthisis, or to their attendants, to prevent the spread of the disease from a focus swarming with the deadly germs?

In the first place, they should all be impressed with the fact that the disease is infectious, and that the agent of infection is a germ that is carried from the diseased individual in the breath to a limited extent, in the excretions from the bowels and sometimes in those from kidneys and from the skin, generally in the vomitus, but in the greatest amount in the sputum; we should further inform them that drying only makes the germs more readily diffused through the atmosphere, that the ordinary process of washing does not destroy their activity; that burning or boiling is the only means of rendering them innocuous, but that as long as they are kept wet they are not disseminated through the air. Now, the practical conclusions that are to be drawn from these scientific facts are, that the underclothing and bedclothing of those suffering from tuberculosis should be frequently changed and the soiled clothing should be boiled for a half hour before being washed; that the excreta should all be passed into vessels containing water, and that these vessels should be rinsed and thoroughly cleaned with boiling water; that the sputum should be expectorated into a cup or other suitable vessel containing water, which is kept covered when not in actual use (if the patient is walking about, a wide-mouthed bottle, well corked, can be carried in the pocket); that the sputum thus collected should be emptied from the vessel three or four times in the twenty-four hours, and *immediately* burned; that the vessel should then be thoroughly cleaned in the same manner as directed for the vessels used for excreta; that the mouth, nose and throat should be cleansed at least twice daily with a mildly alkaline wash; that the table utensils of the patient, including the napkin, should be thoroughly treated with boiling water before being otherwise washed. The well members of the family should not kiss the patient on the mouth; and under no circumstances should any one of them sleep in the same bed with the patient. The patient's room should be thoroughly ventilated and should never be tightly shut up; it should also be as sunny a room as possible. Whatever therapeutic measures may be adopted for the treatment of the case, directions such as the above should be given to the patient, or to some responsible member of the family or to the attendant.

In addition to what is thus done by the physician in the way of special directions to his patients, much — very much — may be accomplished by the State or the municipality through the *competent* inspection of the food and milk supply. Though all cannot be accomplished by either if the other does not coöperate, still, of the two, more can be accomplished by the physician by such careful directions to his private and dispensary cases, than can be by the State if the physician neglects his duty.

Now, what can we do to prevent the occurrence of venereal diseases and so aid in the strengthening and improvement of the race?

Laws may be passed by the State forbidding the existence of brothel-houses, or licensing their existence and placing them under medical and police surveillance; appeals may be made to men and women from a moral standpoint to avoid these places, and refrain from unholy intercourse. These means have all been used, but there is nearly, if not fully, as great prevalence of venereal diseases in those communities where such regulations exist as in those where they do not.

Now, it seems to me that the true way to prevent the occurrence of these diseases is to give instruction to people of all classes as to the nature and serious character of these diseases. To make this instruction effective, it must be given early enough in life to enable young people to avoid exposure when first tempted. Therefore, it seems to me that to boys of fourteen and upwards instruction should be given in the physiology of reproduction, and, at the same time, the sacredness of marriage should be impressed upon them. Then it should be plainly stated to them that some men and women put themselves on a level with, or even on a lower level than, the beasts by indulging in indiscriminate sexual intercourse; that these women are known as whores, and that they and the men who associate with them are despised by all decent people; but that, unfortunately for the cause of high morality, sometimes some such people are tolerated by their fellows for the sake of other good qualities which they may possess; however, that, sooner or later, nearly all who indulge in such intercourse are afflicted with either gonorrhea or syphilis, or both; that rarely, if ever, is either of these diseases completely cured. Then all the evil results of syphilis should be vividly portrayed to them, illustrated by plates taken from real life.

The same thing should be done in regard to gonorrhea.

A similar course of lectures should be given to the young girls of all walks of life by some of the women physicians.

Finally, it should be impressed upon them that no one, who has ever had syphilis, has any right *ever* to marry, for there are cases on record, in which, after a lapse of twenty, thirty and even fifty years from the time of the original lesion, tertiary symptoms have developed, although no secondary symptoms had shown themselves during the last three-quarters of the period, and the patients had deemed themselves well; for should such a one marry and beget children, these children would have a syphilitic taint that would show itself either in real syphilitic lesion or in an enfeebled constitution. Nor should any one who has ever had gonorrhea ever marry, if there remains the slightest trace of the original disease or any of its sequelæ—and it is very, very rare that some such trace does not remain. The danger of such a one's infecting his wife and the terrible results of gonorrhea in women, should be plainly set forth.

Moreover, it is our duty as physicians and good citizens looking to the welfare of humanity and the progress of true civilization, to advise any, who may consult us as to the propriety of his marrying, according to the principles just stated.

Fellows of the Academy, let us be among the pioneers in putting to practical use the results of the discoveries of the bacteriologists and thus hastening the advent of the medicine of the future—preventive medicine!

